# Bayesian Inversion for Geoacoustic Parameters in Shallow Sea †

**DOI:** 10.3390/s20072150

**Published:** 2020-04-10

**Authors:** Guangxue Zheng, Hanhao Zhu, Xiaohan Wang, Sartaj Khan, Nansong Li, Yangyang Xue

**Affiliations:** 1 College of Underwater Acoustic Engineering, Harbin Engineering University, Harbin 150001, China; zgx1057@hrbeu.edu.cn (G.Z.); wangxiaohan@hrbeu.edu.cn (X.W.);; 2 Institute of Marine Science and Technology, Zhejiang Ocean University, Zhoushan 316022, China; 3 State Key Laboratory of Acoustics, Chinese Academy of Sciences, Beijing 100190, China; 4 Acoustic Science and Technology Laboratory, Harbin Engineering University, Harbin 150001, China; 5 Key Laboratory of Marine Information Acquisition and Security Industry and Information Technology, Harbin Engineering University, Harbin 150001, China; 6 Institute of Naval Architecture and Mechanical-electrical Engineering, Zhejiang Ocean University, Zhoushan 316022, China

**Keywords:** underwater acoustic sensor, Bayesian inversion, shallow sea, geoacoustic parameters

## Abstract

Geoacoustic parameter inversion is a crucial issue in underwater acoustic research for shallow sea environments and has increasingly become popular in the recent past. This paper investigates the geoacoustic parameters in a shallow sea environment using a single-receiver geoacoustic inversion method based on Bayesian theory. In this context, the seabed is regarded as an elastic medium, the acoustic pressure at different positions under low-frequency is chosen as the study object, and the theoretical prediction value of the acoustic pressure is described by the Fast Field Method (FFM). The cost function between the measured and modeled acoustic fields is established under the assumption of Gaussian data errors using Bayesian methodology. The Bayesian inversion method enables the inference of the seabed geoacoustic parameters from the experimental data, including the optimal estimates of these parameters, such as density, sound speed and sound speed attenuation, and quantitative uncertainty estimates. The optimization is carried out by simulated annealing (SA), and the Posterior Probability Density (PPD) is given as the inversion result based on the Gibbs Sampler (GS) algorithm. Inversion results of the experimental data are in good agreement with both measured values and estimates from Genetic Algorithm (GA) inversion result in the same environment. Furthermore, the results also indicate that the sound speed and density in the seabed have fewer uncertainties and are more sensitive to acoustic pressure than the sound speed attenuation. The sea noise could increase the variance of PPD, which has less influence on the sensitive parameters. The mean value of PPD could still reflect the true values of geoacoustic parameters in simulation.

## 1. Introduction

In shallow sea environments, sound propagation is usually constrained and significantly affected by the geoacoustic parameters such as density, sound speed, and sound speed attenuation in the sea bottom [1]. The recent importance of study on acoustic problems in shallow sea has magnified the requirement for correct geoacoustic parameter estimates [2]. To obtain the sea bottom geoacoustic parameters, there are two methods. One uses the kinds of samplers to get the sea bottom properties directly, the other uses the acoustic signals to invert for these properties based on the sea bottom information implicitly carried in the signals, known as geoacousitc inversion. Furthermore, compared to the method of direct measurement, using acoustic signals to invert geoacoustic parameters are more efficient, and have attracted wide research interest over the past decade [3,4,5,6,7,8].

**Figure 1:**
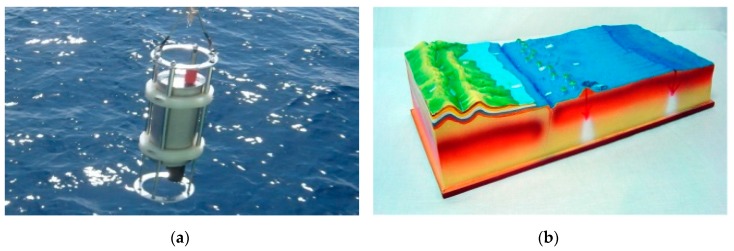
(**a**) On-site measurement, (**b**) Geoacoustic parameters inversion.

Nowadays, there are two ways to obtain marine environmental parameters. One is direct measurement, including on-site measurement and post-sampling analysis. The other method is acoustic inversion. Figure 1a,b show the on-site measurement and geoacoustic parameters inversion respectively. Compared with direct measurement, the acoustic inversion method can obtain the acoustic parameters quickly and efficiently, and avoid the waste of manpower and material resources.

Past studies revealed that geoacoustic inversion is mainly based on data measured by Vertical Linear Array (VLA) or Horizontal Linear Array (HLA). The sea bottom was usually regarded as a liquid medium, which only considers three geoacoustic parameters, namely, the compression wave (P-wave) speed, the density of the sea bottom, and P-wave speed attenuation. Most of these studies used the optimize methods such as Genetic Algorithm (GA) and Simulated Annealing (SA), etc., to solve the cost functions and thus get the optimal solution. But in practical application, a VLA or HLA carries a high computational cost, and the accuracy of the measured data can be affected greatly by the array shape, which is influenced by ocean currents and storms [9]. Therefore, more and more geoacoustic inversion based on low-frequency sound propagation characteristics have been proposed, and these methods greatly reduce the cost and complexity for recording systems in measurement [10,11,12,13]. However, the sea bottom was still set like a liquid medium. It has been proved that, when the low-frequency acoustic signal propagating in a shallow sea, the influence of shear wave (S-wave) in the bottom cannot be ignored [14], the sea bottom should be set as an elastic medium.

In addition, the geoacousitc inversion is typically a highly nonlinear, multi-parameter, and multi-optima inversion problem. There is usually not a single optimal solution due to the measurement and calculation errors. The inversion results of traditional optimize methods are all prone to being trapped by local minima, can only determine the best-fit model and not provide quantitatively nonlinear uncertainty estimation of the awaiting inversion parameters. In recent years, the Bayesian inversion method underwent considerable development and significantly contributed to estimating bottom properties and their uncertainties based on a Bayesian formulation [15,16,17,18,19]. The Bayesian inversion method is a global optimization algorithm based on the probability theory, applied mathematics, and optimization theory. It can efficaciously estimate Maximum Posterior Probability (MAP) model parameters, and then qualitatively and quantitatively analyze the uncertainty of parameter inversion results from a statistical perspective.

To close the gap, an effort has been made in this study to develop an inversion method for geoacoustic parameters by using a single acoustic sensor based on Bayesian theory. The significant impact from the elastic sea bottom on the low-frequency sound propagation in the shallow sea can be used to inference the geoacoustic parameters. According to Gibbs Sampler (GS) algorithm, the Posterior Probability Densities (PPDs) of five geoacoustic parameters, including the P-wave speed, S-wave speed, the density of the sea bottom, P-wave speed attenuation, and S-wave speed attenuation are given as the inversion results. Further, the effectiveness of this method is verified by the simulation analysis and an anechoic tank experiment. Before verification, the sound propagation characteristics were analyzed, which corresponded to the inversion result. For further confirmation, the Transmission Loss (TL) and the time domain diagram were compared, respectively, which include the measured results and the simulation results. The comparison shows that measured results are consistent with simulation results, which proves the reliability of the inversion method.

This paper is organized as follows: the prediction model of the underwater acoustic-field is described in Section 2 based on Pekeris waveguide. In Section 3.1 the inversion method based on Bayesian theory is introduced. The cost function is established by measuring sound pressure and the prediction sound pressure and presented in Section 3.2. The analysis of sound propagation characteristics is given in Section 4.1 followed by the inversion parameter’s sensitivity and inversion results of simulation data (simulated in the noiseless and noisy sea environment) in Section 4.2 and Section 4.3 respectively. The scaling experiment carried out in the laboratory is introduced in Section 5.1 followed by the procedure of the scaling experiment in Section 5.2. The inversion results of the scaling experiment and verification of the results are presented in Section 5.3 and Section 5.4, respectively. Finally, conclusions are given in Section 6.

## 2. Prediction Modeling of a Shallow Sea Acoustic Field

**Figure 2:**
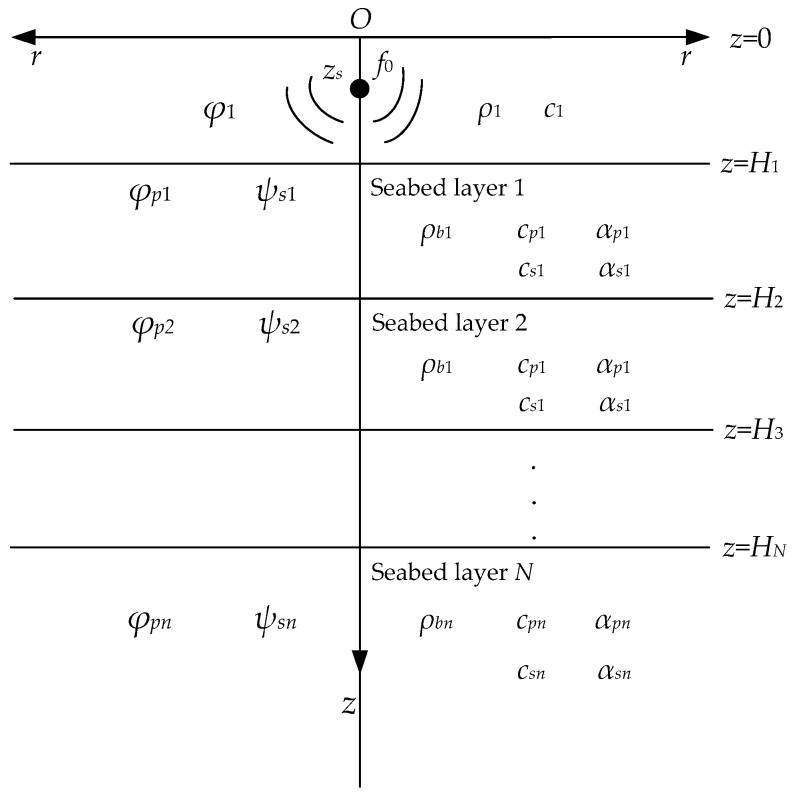
A layered shallow sea waveguide model.

The S-wave of the seabed has a significant impact on the propagation of low-frequency acoustic signals in the shallow sea environments. The environment model in this study is developed based on Pekeris waveguide [20], and the seabed is regarded as an *N*-layered elastic medium. A schematic diagram of the waveguide model is shown in Figure 2. The *z*-axis indicates the depth of the sea, and the *r*-axis represents the propagation direction of acoustic signals. As shown in the figure, *φ*_1_, *φ_p__n_*, and *ψ_s__n_* are the displacement potential function in the water column, P-wave displacement potential function, and S-wave displacement potential function in the layered seabed, respectively. *ρ*_1_ and *c*_1_ are the density and sound speed in the water column. *ρ_b__n_*, *c_p__n_*, and *c_s__n_* are the density, compression wave (P-wave) speed, and shear wave (S-wave) speed, respectively, of the *n* layer of seabed, *α_p__n_* and *α_s__n_* represent the attenuation of P-wave and S-wave, respectively; these five parameters in each layer of seabed are the expected inversion objects in this study. Moreover, *z_s_* is the depth of the point acoustic source, *z* = 0 and *z* = *H* are set as the sea surface and seabed in the model.

According to the wave theory, in the frequency domain, the displacement potential functions of each layer in Figure 2 satisfy the following equations:

(1) $$\frac{1}{r}\frac{\partial }{\partial r}\left(r\frac{\partial \varphi_{1}}{\partial r}\right)+\frac{\partial^{2}\varphi_{1}}{\partial z^{2}}+k_{1}^{2}\varphi_{1}=-4\pi \delta \left(r,z-z_{s}\right),\;0\leq z<H_{1}$$

(2) $$\{\begin{array}{c} \frac{1}{r}\frac{\partial }{\partial r}\left(r\frac{\partial \varphi_{pn}}{\partial r}\right)+\frac{\partial^{2}\varphi_{pn}}{\partial z^{2}}+k_{pn}^{2}\varphi_{pn}=0\\ \nabla \times \nabla \times \psi_{sn}-k_{sn}^{2}\psi_{sn}=0 \end{array},\;H_{n}\leq z<H_{n+1}\;,\;n\geq 1$$

(3) $$\{\begin{array}{c} \frac{1}{r}\frac{\partial }{\partial r}\left(r\frac{\partial \varphi_{pN}}{\partial r}\right)+\frac{\partial^{2}\varphi_{pN}}{\partial z^{2}}+k_{pN}^{2}\varphi_{pN}=0\\ \nabla \times \nabla \times \psi_{sN}-k_{sN}^{2}\psi_{sN}=0 \end{array},\;z\geq H_{N}$$

where *δ*(*r*, *z*) is the source function, *k_m__n_*=*ω*/*c_m_* (*m*=1,*p*,*s* and *n*=1…*N*) is the wave number of each layer and *ω*=2π*f*_0_ is the angular frequency of the point acoustic source at *f*_0_.

The general solutions of Equations (1)–(3) can be expressed as follows:

(4) $$\varphi_{1}\left(r,z,\omega \right)=\int_{0}^{\infty }Z_{1}\left(z,\xi ,\omega \right)J_{0}\left(\xi r\right)\xi d\xi ,\;Z_{1}\left(z,\xi ,\omega \right)=\{\begin{array}{cc} A\cdot \sin\beta z & 0\leq z<z_{s}\\ B\cdot \sin\beta_{1}z+C\cdot \cos\beta_{1}z & z_{s}<z<H_{1} \end{array}$$

(5) $$\{\begin{array}{c} \varphi_{pn}\left(r,z,\omega \right)=\int_{0}^{\infty }Z_{pn}\left(z,\xi ,\omega \right)J_{0}\left(\xi r\right)\xi d\xi \\ \psi_{sn}\left(r,z,\omega \right)=\int_{0}^{\infty }Z_{sn}\left(z,\xi ,\omega \right)J_{1}\left(\xi r\right)\xi d\xi \end{array},\;\{\begin{array}{c} Z_{pn}\left(z,\xi ,\omega \right)=P_{n}^{up}e^{-i\beta_{pn}z}+P_{n}^{down}e^{i\beta_{pn}z}\\ Z_{sn}\left(z,\xi ,\omega \right)=S_{n}^{up}e^{-i\beta_{sn}z}+S_{n}^{down}e^{i\beta_{sn}z} \end{array}\;H_{n}\leq z<H_{n+1}\;,\;1\leq n<N$$

(6) $$\{\begin{array}{c} \varphi_{pN}\left(r,z,\omega \right)=\int_{0}^{\infty }Z_{pN}\left(z,\xi ,\omega \right)J_{0}\left(\xi r\right)\xi d\xi \\ \psi_{sN}\left(r,z,\omega \right)=\int_{0}^{\infty }Z_{sN}\left(z,\xi ,\omega \right)J_{1}\left(\xi r\right)\xi d\xi \end{array},\;\{\begin{array}{c} Z_{pN}\left(z,\xi ,\omega \right)=P_{N}e^{i\beta_{pN}z}\\ Z_{sN}\left(z,\xi ,\omega \right)=S_{N}e^{i\beta_{\mathrm{sN}}z} \end{array}z\geq H_{N}$$

where $\beta_{mn}=\sqrt{k_{mn}^{2}-\xi^{2}}$ (*m* = 1,*p*,*s* and *n* = 1…*N*), *ξ* is the horizontal wavenumber, *J*_0_ and *J*_1_ are the first order and second-order Bessel functions, respectively, and *A*, *B*, *C*, *P_n_* and *S_n_* are the indeterminate coefficients of the potential functions in each layer, the high mark ‘up’ and ‘down’ are used to representative the upgoing waves and downgoing waves in these potential functions. In the water column, the relationship between sound pressure *p* and potential function *φ*_1_ is $p=\rho_{1}\omega^{2}\varphi_{1}$.

Based on the point source condition, the boundary conditions on the fluid/solid interface for *H*_1_ (continuous normal displacement, continuous normal stress, and zero tangential stress) and *H_n_*_+1_(continuous normal displacement, continuous normal stress, continuous tangential displacement, and continuous tangential stress), the relationship between these coefficients can be written as a (4*N*+1)th order matrix equations, which is shown in Equation (7).

(7) $${\left(a_{ij}\right)}_{\left(4N+1\right)\times \left(4N+1\right)}\cdot {\left(b_{ij}\right)}_{\left(4N+1\right)\times 1}={\left(c_{ij}\right)}_{\left(4N+1\right)\times 1}\;N\geq 1$$

The various indeterminate coefficients, such as *A*, *B*, *C*, *P_n_* and *S_n_*, can be solved by (*b_ij_*) _(4*N*+1) ×1_ = [(*a_ij_*)_(4*N*+1) ×__(4*N*+1)_]^−1^. (*c_ij_*) _(4*N*+1) ×1_. Substituting *b_ij_* into Equations (4)–(6) further result in the potential functions in each layer. When replacing the coefficients in Equations (4)–(6), the expressions of displacement potential function in Figure 2 can be obtained. The expressions of sound pressure *p* in the water column, as the study object in inversion, can also be obtained with Equation (8).

(8) $$p\left(r,z,\omega \right)=\rho_{1}\omega^{2}\int_{0}^{\infty }Z_{1}\left(z,\xi ,\omega \right)J_{0}\left(\xi r\right)\xi d\xi$$

(9) $$p\left(r_{j},z,\omega \right)=\frac{S\left(\omega \right)\rho_{1}\omega^{2}\Delta \xi }{\sqrt{2\pi r}}e^{i\left(\xi_{\min}r_{j}-\frac{\pi }{4}\right)}\sum_{l=0}^{N_{s}-1}\left[Z_{1}\left(z,\xi_{l}\right)e^{ir_{\min}l\Delta \xi }\sqrt{\xi_{l}}\right]e^{i\frac{2\pi lj}{N_{s}}}$$

*ξ_l_* = *ξ*_min_ + *l*Δ*ξ*, *l* = 0, 1, 2, …, (*N_S_*−1), *r_j_* = *r*_min_ + *j*Δ*r*, *j* = 0, 1, 2, …, (*N_S_*−1), Δ*r* · Δ*ξ* = 2π/*N_S_*.

For the integral term of sound pressure in Equation (8), the Fast Field Method (FFM) considers the impact of lateral waves in the calculation and can measure the sound pressure easily and reliably [21]. It is chosen to describe the *p* for inversion in this study. It first discretizes the horizontal wavenumber *ξ* and the propagation distance *r* of Equation (9), and then the integral term is solved directly by using Fast Fourier Transform (FFT) [22].

## 3. Inversion Method

### 3.1. Bayesian Inversion Theory

Set the experiment measured data vector as **d** with elements *d_i_*, and let **m** represent the model vector composed of the awaiting inversion geoacoustic parameters *m_i_*. Both *d_i_* and *m_i_* are considered random variables that are related via Bayes’ rule [23]

(10) P(m|d)=P(d|m)P(m)/P(d)

The *P*(**m**|**d**) is the posterior probability density (PPD). *P*(**d**|**m**) is the conditional probability density function (PDF) of **m** under given **d**, *P*(**m**) is the prior PDF of **m** and representing the available parameter information independent of the data **d**, *P*(**d**) is the PDF of **d**. As the *P*(**d**) is independent of **m**, and the *P*(**d**|**m**) can be regarded as the likelihood function *L*(**d**|**m**) for the measured data [23], the Equation (10) can be written as:

(11) P(m|d)∝L(m)P(m)

The *L*(**m**) is determined by the form of the data and the statistical distribution of the data errors, including both measurement and model errors. Considering that it is difficult to obtain an independent estimate of the error statistics in practice, the assumption of unbiased Gaussian errors is used in processing, the form of the likelihood function is

(12) L(m)=P(d|m)∝exp[−E(m)]

where *E(***m***)* is the cost function. After normalizing

(13) $$P(m|d)=\frac{\exp[-E(m)]P(m)}{\int \exp[-E(m^{\prime })]P(m^{\prime })dm^{\prime }}$$

The integration domain spans an *M*-dimensional parameter space, *M* is the number of awaiting inversion parameters.

In Bayesian theory, the posterior probability density (PPD) of **m** can be regarded as the inversion results. To interpret the *M*-dimensional PPD requires us to estimate the properties of the parameter value, uncertainties, and inter-relationships, such as the MAP model $\hat{m}$, mean model $\bar{m}$ and marginal probability distributions *P*(*m_i_*|**d**). These properties are respectively defined as:

(14) $$\hat{m}={\mathrm{Arg}}_{\max}\{P(m|d)\}$$

(15) $$\bar{m}=\int m^{\prime }P(m^{\prime }|d)dm^{\prime }$$

(16) $$P(m_{i}|d)=\int \delta (m_{i}-m_{i}{}^{\prime })P(m^{\prime }|d)dm^{\prime }$$

### 3.2. Cost Function

A sufficient cost function is necessary to evaluate the geoacoustic parameters using the Bayesian inversion method. Via Bayes’ rule, the cost function is developed based on the likelihood function *L*(**m**)under the assumption of the Gaussian data errors [24].

(17) $$L(m)=\prod_{f=1}^{F}\frac{1}{\pi^{K}\left|C_{m}^{f}\right|}\exp\left\{-{[p_{\mathrm{mea}}^{f}-p_{\mathrm{pre}}^{f}(m)]}^{T}{\left(C_{m}^{f}\right)}^{-1}[p_{\mathrm{mea}}^{f}-p_{\mathrm{pre}}^{f}(m)]\right\}$$

where $p_{\mathrm{mea}}^{f}(m)$ presents the measured sound pressure data at *K*receive positions by a single sensor under the *f*th frequency, $p_{\mathrm{pre}}^{f}(m)$ and $C_{m}^{f}$ respectively present the model prediction sound pressure data and the covariance matrix under the same condition.

Consider the model prediction sound pressure $p_{\mathrm{pre}}^{f}(m)$ can be expressed as:

(18) $$p_{\mathrm{pre}}^{f}(m)=A^{f}e^{i\theta_{f}}p_{\mathrm{FFM}}^{f}(m)$$

where $p_{\mathrm{FFM}}^{f}(m)$ is the sound pressure computed via the FFM, *A^f^* and *θ^f^* is the magnitude and phase of the unknown complex source at each frequency. To remove the dependence on *A^f^* and *θ^f^* by setting the $\partial L(m)/\partial A^{f}=\partial L(m)/\partial \theta^{f}=0$ [25], the maximum likelihood solution of the source is

(19) $$A^{f}e^{i\theta^{f}}=\frac{{\left[p_{\mathrm{FFM}}^{f}(m)\right]}^{*}p_{\mathrm{mea}}^{f}}{{\left|p_{\mathrm{FFM}}^{f}(m)\right|}^{2}}$$

where * denotes the conjugate transpose.

Ignoring the spatial correlation of the data, the common approximation of diagonal covariance $C_{m}^{f}=v^{f}I$, where *v^f^* is the unknown variance at the *f*th frequency and **I** is the identity matrix, the *L*(**m**)can be simplified written as:

(20) $$L(m)=\prod_{f=1}^{F}\frac{1}{{\left(\pi \nu^{f}\right)}^{K}}\exp\left[-\frac{B^{f}(m){\left|p_{\mathrm{mea}}^{f}\right|}^{2}}{\nu^{f}}\right]$$

where Equation (20) represents the normalized Bartlett disqualification:

(21) $$B^{f}(m)=1-\frac{{\left|{\left[p_{\mathrm{FFM}}^{f}(m)\right]}^{*}p_{\mathrm{mea}}^{f}\right|}^{2}}{{\left|p_{\mathrm{mea}}^{f}\right|}^{2}{\left|p_{\mathrm{FFM}}^{f}(m)\right|}^{2}}$$

To obtain a maximum-likelihood estimate of the data variance by setting the $\partial L(m)/\partial v^{f}=0$, the maximum likelihood solution of the variance estimate is:

(22) $${\hat{\nu }}_{f}=\frac{B^{f}(m){\left|p_{\mathrm{mea}}^{f}\right|}^{2}}{K}$$

Substituting Equation (22) into Equation (20) and Equation (12), the cost function *E*(**m**) becomes

(23) $$E(m)=K\sum_{f=1}^{F}\mathrm{In}\left[B^{f}(m){\left|p_{\mathrm{mea}}^{f}\right|}^{2}\right]$$

where the Maximum Posterior Probability model $\hat{m}$ for measured data with unknown variances can be found by minimizing the *E*(**m**), using a global optimization scheme.

**Figure 3:**
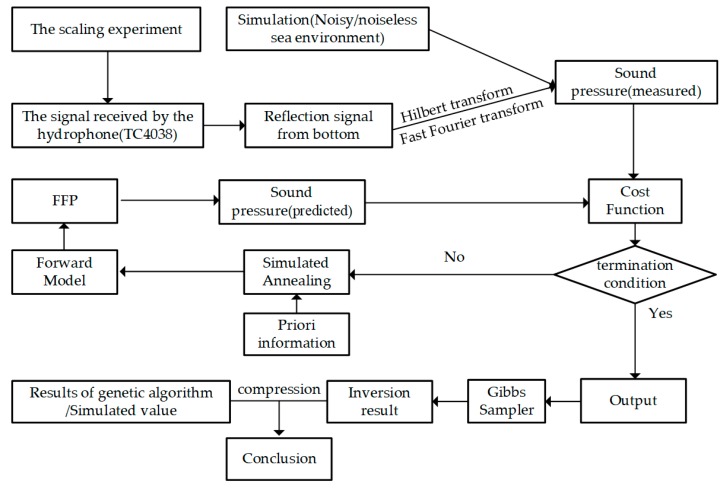
The diagram of the research.

As can be seen from Figure 3, in the research, the simulated annealing (SA) is used to optimize the $\hat{m}$ of expecting geoacoustic parameters from Equation (23). The Gibbs sampler (GS) algorithm is applied to compute the PPD moments as defined in Equations (10)–(13), and the sample model is accumulated by SA at temperature *T* = 1 [26]. In Section 5, the genetic algorithm (GA) is also used to optimize the $\hat{m}$, which is regarded as a compression to verify the inversion method proposed in this paper. For SA settings, the starting *T_0_* = 100, cooling rate *ξ* = 0.99, the number of samples n = 10000, and the type of perturbation is non-uniform. For GA settings, the population of each generation is pop = 1000, the type of coding is binary coding, the maximum generation is 100, the type of selection is roulette wheel selection, the cross-over rate is 0.95, and the mutation rate is 0.05. GS settings corresponded to the SA settings. A block diagram of the research is shown in Figure 3.

## 4. Simulation Results

Considering the shallow sea environment, where the seabed is regarded as a semi-infinite elastic medium, the following research is conducted based on the model with a 1-layered elastic bottom. In this model, the inversion objects are five geoacoustic parameters, namely, the P-wave speed *c_p_*, S-wave speed *c_s_*, the attenuation coefficient for the two speeds *α_p_* and *α_s_*, and seabed density *ρ_b_*. The true values of waveguide parameters in the simulation are presented in Table 1.

### 4.1. The Analysis of the Acoustic Propagation Characteristics

In a simulation, the transmission loss (TL) was chosen to reveal the change of acoustic propagation characteristics, and the TL is defined as Equation (24).

(24) $$\mathrm{TL}=20\mathrm{lg}\left|\frac{p\left(r_{j},z,\omega \right)}{{p_{ref}|}_{r=1m}}\right|,\;p_{ref}=\frac{e^{ikr}}{r}$$

where the *p*(*r_j_*, *z*, *ω*) was calculated by Equation (22).

**Figure 4:**
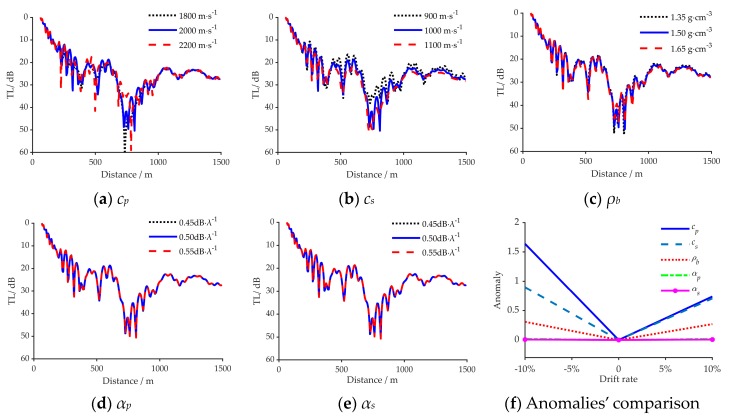
The influence of the five geoacoustic parameters on acoustic propagation, (**a**)-(**e**) corresponds to the *c_p,_ c_s,_ ρ_b,_* *α_p_* and *α_s_* respectively, (**f**) reveals the comparison of TLs’ anomalies when the value of each parameter was changed.

Figure 4 reveals the comparison results of the TL under different geo-acoustic parameters in the simulation. Figure 4a to Figure 3e show the influence of *c_p,_ c_s,_ ρ_b,_* *α_p_*, and *α_s_* on acoustic propagation separately. The changing values of each parameter were set to the simulation true value deviation ±10%; when one of the above parameters changed, the other parameters remained fixed. In Figure 4a–e, the solid blue line represents the calculation results under the simulated true values, where the dotted black line and the dashed red line indicate the calculation results under the changing values. Figure 4f revealed the comparison of TLs’ anomalies when the value of each parameter was changed, and the change bounds were set between −10% to +10% of the true value [27].

It can be deduced from Figure 4a–e that the change of five parameters has a different impact on the acoustic propagation characteristics. Keeping the other simulation conditions fixed, the Transmission Loss (TL) changes the most when the *c_p_* and *c_s_* are varied. When *ρ_b_* changes, the variation in the Transmission Loss (TL) is relatively prominent. When the *α_p_* or *α_s_* is changed, the variation in the Transmission Loss (TL) is the least obvious. The anomaly values of the five parameters in Figure 4f reveal that, in the discus bounds, these parameters are in the descending order of degree of influence on TL are *c_p_*, *c_s_*, *ρ_b_*, *α_p_*, and *α_s_*. In this situation, the influence order of degree of five parameters on acoustic propagation characteristics can be preliminarily summarized as: *c_p_*>*c_s_*>*ρ_b_*>*α_p_*>*α_s_*.

### 4.2. The Analysis of the Inversion Parameters’ Sensitivity

**Figure 5:**
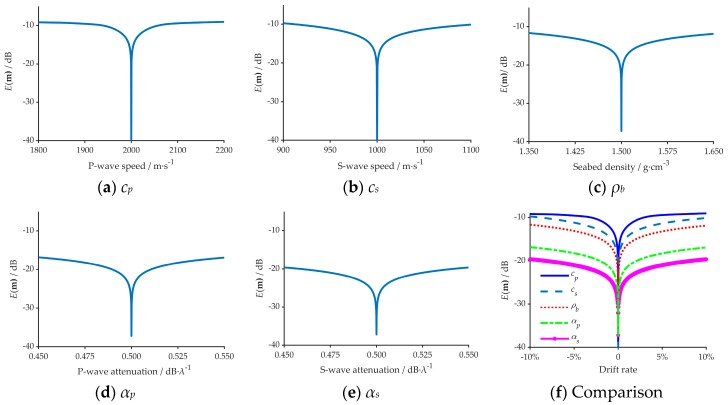
The sensitivity of the cost function (*E*(**m**)) for the five geoacoustic parameters. (**a**) to (**e**)corresponds to the *c_p,_ c_s,_ ρ_b,_* *α_p_*, and *α_s_*, respectively. (**f**) corresponds to the comparison of five parameter’s influence on *E*(**m**).

Using the simulation environmental conditions, as mentioned above, Figure 5a–e respectively represent the numerical variations of the cost function *E*(**m**) with the change of a single parameter. In each search bound of the parameter, *E*(**m**) touches the minimum value only at the true simulated value of the parameter, which can avoid the impact of the local optimum solution on the optimization of the cost function in the subsequent algorithm. Nonetheless, with the change of those five parameters, the range of *E*(**m**) variation is different. It can be seen from Figure 5f, that within the search bounds of five parameters, the parameters are in the descending order of degree of influence on *E*(**m**) are *c_p_*, *c_s_*, *ρ_b_*, *α_p_* and *α_s_*. Therefore, the influence order of the degree of five geoacoustic parameters on *p*(*r*, *z*) can be defined *as*: *c_p_* > *c_s_* > *ρ_b_* > *α_p_* > *α_s_*. This further verifies our results discussed in Section 4.1.

### 4.3. The Inversion Results

In this section, based on the research basis in Section 4.1 and Section 4.2, the feasibility and reliability of the inversion method are discussed with simulation. The focus of discussion is the application effect of the inversion method in noisy shallow sea environment. It can be seen from the literature [28], that the energy spectrum level of the noise in shallow sea could be −40 dB (0dB@1Pa), the energy spectrum level in simulation is set as −40 dB.

**Figure 6:**
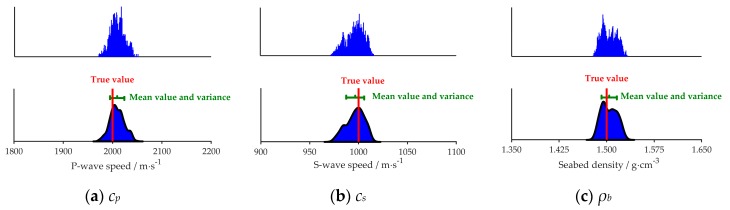

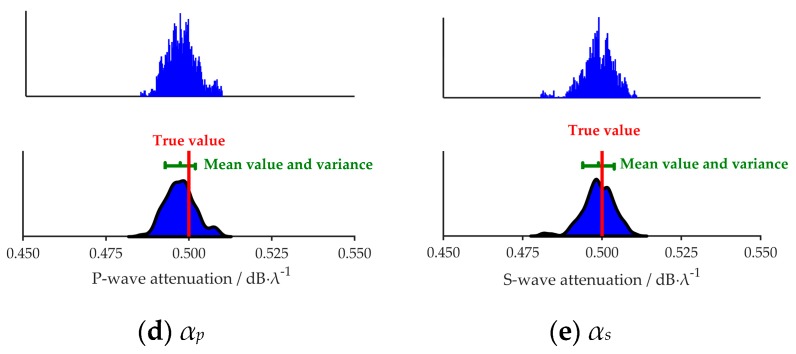
Five geo-acoustic parameters’ Posterior Probability Density (PPD) in the noiseless sea environment. The red lines mean the true value of each parameter in simulation. The green segments represent the mean value of the inversion results and their variance.

**Figure 7:**
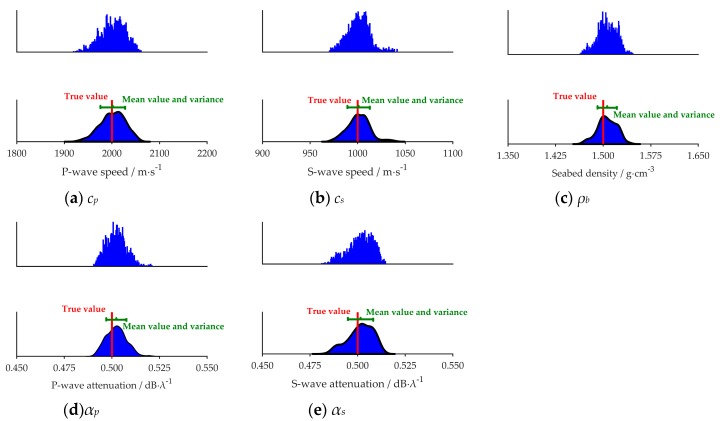
Five geo-acoustic parameters’ PPD in the noisy sea environment. The red lines mean the true value of each parameter in simulation. The green segments represent the mean value of the inversion results and their variance.

Figure 6 and Figure 7 show the PPDs of the five geoacoustic parameters in the noiseless and noisy sea environment respectively. In each figure, the vertical axis represents the probability, and true value means the simulated value indicated by the red line. The mean value and variance of inversion results are represented by the green segment in which the mean value is shown at the middle of the segment. The length of the green segment reflects the variance of each parameter.

It can be inferred that, in simulation, the maximum value of the probability density of each parameter is near to its true value, and the sharpness of the probability density curve shows the sensitivity of the cost function to each parameter: *c_p_*, *c_s_* > *ρ_b_* > *α_p_* and *α_s_* (Figure 6 and Figure 7). Further, the results are lining with the findings presented in Section 4.1 and Section 4.2. Comparing Figure 6 and Figure 7, we can find that for the same parameter the PPD in Figure 7 is wider.

**Figure 8:**
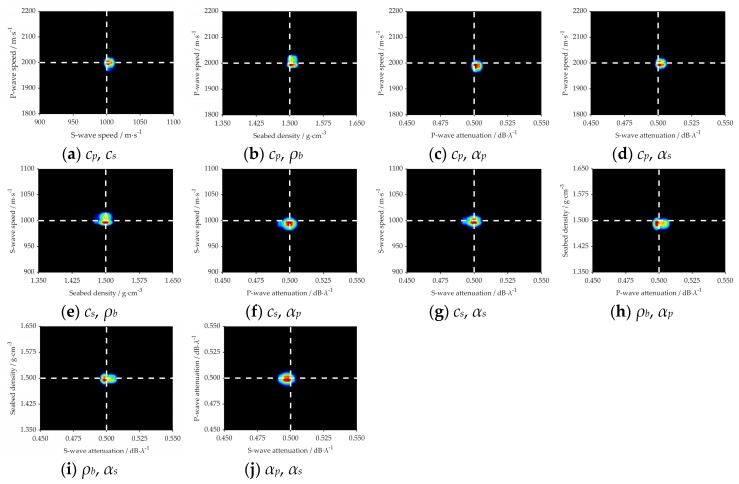
2D marginal PPDs between different parameters in the noiseless sea environment. White dashed lines mark the true values of each parameter in the simulation.

**Figure 9:**
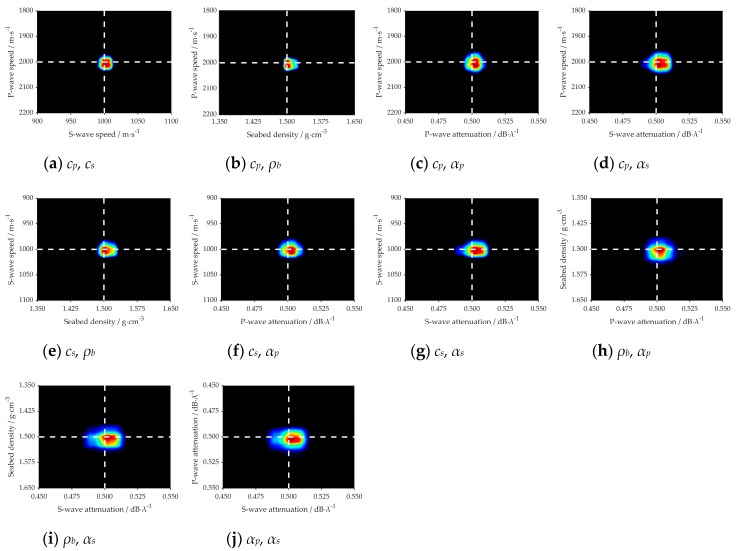
2D marginal PPDs between different parameters in the noisy sea environment. White dashed lines mark the true values of each parameter in the simulation.

The marginal probability distribution between two parameters is presented in Figure 8 and Figure 9, which shows the uncertainty of different parameters. The true value of each parameter in the simulation is indicated with a white dashed line. It can be seen clearly from the figures that all the true values are close to the highest probability of PPD.

For the parameters *c_p_*, *c_s_* and *ρ_b_*, the inversion results are similar in the two different marine environments, such as Figure 6a–c, Figure 7a–c, Figure 8a–c,e,f and Figure 9a–c,e,f. It is summarized that noise has less effect on the inversion results of sensitive parameters.

To clearly figure out the effect of noise on inversion results, Table 2 gives various inversion results between above two conditions. The value of each parameter is within the mean ± variance in the table. We can easily see that in the noiseless environment, the mean values of the inversion results are closer to the true value, and the variances are smaller. The sea noise could increase the variance of inversion results, but the mean of inversion results still could reflect the true values in simulation.

**Figure 10:**
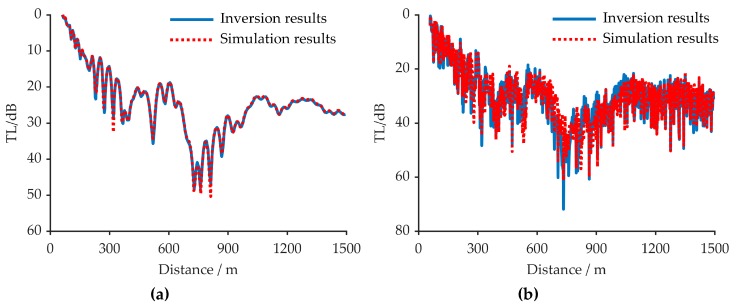
The verification of simulation results. The solid blue line means the Transmission Loss (TL) measured in the simulation and the dashed red line represents the TL simulated by inversion result. (**a**) the comparison of TL in the noiseless sea environment; (**b**) the comparison of TL in the noisy sea environment.

Figure 10 shows the comparison of TLs in the different sea environment. As can be seen from Figure 10, the inversion results are both consistent with the simulation results. The results in Figure 10 and Table 2 proved that no matter whether it is applied to a noiseless or noisy sea environment, the developed inversion method is reliable.

## 5. Results of Measured Data

### 5.1. Introduction to the Scaling Experiment

Suppose in a shallow sea waveguide, the various acoustic parameters (sound speed, sound speed attenuation and density) remain unchanged, the geometric parameters for the waveguide are scaled down by *N* times whereas the sound frequency is scaled up by *N* times, as *H′* = *H*/*N*, *z_s_* = *z*/*N*, *z′* = *z*/*N*, *r′* = *r*/*N*, and *f′* = *Nf*. In this case, the relationship between the original pressure *p* and the scaled pressure *p*′ can be obtained: *p*′(*r*′, *z*′, *ω*′) = *Np*(*r*, *z*, *ω*), and both the fluctuation and distribution of the original acoustic field remain unchanged in the scaled acoustic field [29].

**Figure 11:**
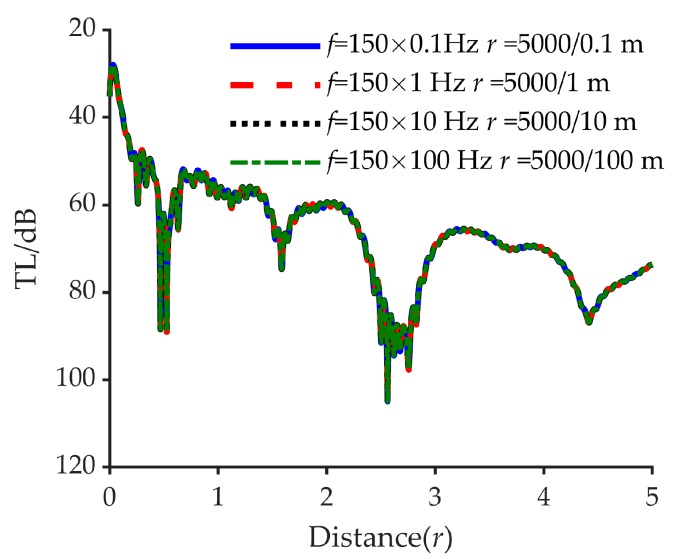
Transmission losses under the four scaling conditions. The solid blue line represents the frequency 150 × 0.1 Hz, the dashed red line represents the frequency 150 × 1 Hz, the dotted black line represents the frequency 150 × 10 Hz, and the dashed dotted green line represents the frequency 150 × 100 Hz.

Figure 11 displays the transmission losses under four scaling conditions, namely *N* = 0.1, 1, 10, 100. The corresponding sound frequency and water depth are 150 × 0.1 Hz, 150 × 1 Hz, 150 × 10 Hz, 150 × 100 Hz, and 100/0.1 m, 100/1 m, 100/10 m and 100/100 m, respectively. The acoustic source depth *z_s_* and receive depth *z_r_* are 20/0.1 m, 20/1 m, 20/10 m, 20/100 m and 10/0.1 m, 10/1 m, 10/10 m, 10/100 m respectively. The values of various acoustic parameters are set to be the same as the true value in Section 4.

The results of the theoretical analysis and the simulation indicate that the propagation of a high-frequency underwater acoustic signal in small-sized acoustic fields such as in a tank can be used to simulate the propagation of low-frequency underwater acoustic signals in large-sized environments such as the ocean. Thus, we carried out a scaling experiment, keeping in mind that the experiments performed in a tank in the laboratory have the advantages of stable environmental conditions, simple equipment distribution, and low cost.

### 5.2. Procedure and Data Processing of the Scaling Experiments

**Figure 12:**
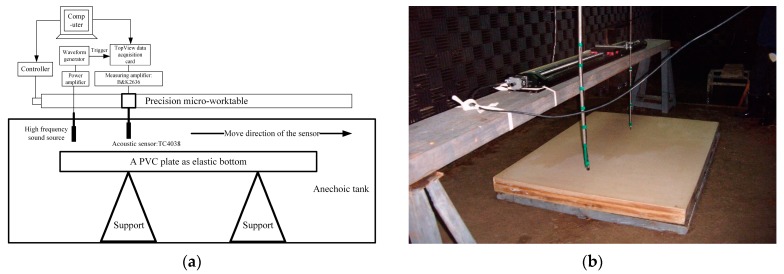
(**a**) Experimental measurement system, (**b**) The movable micro-worktable for measurement equipment.

The experimental data were eventually utilized to validate the above-mentioned inversion method. The automatic measurement system is shown in Figure 12a,b. The experiment used a Poly Vinyl Chloride (PVC) slab (size: 1.53 m × 1.1 m × 0.105 m) to simulate the semi-infinite elastic bottom. A high-frequency underwater sound wave is transmitted by a source at a fixed position, and the wave is received by a single acoustic sensor at different positions with equal lengths. To increase the accuracy of the measurement, the acoustic sensor was fixed on a precision micro-worktable. With the micro-worktable, the acoustic sensor can move 2 mm every time with an error of less than 20 um, and a computer was used to control the micro-worktable and acquire data. When the measurement finishes at one position, the micro-worktable automatically takes the acoustic sensor to the next position according to the desired direction.

Table 3 shows the measurement parameters used in the experiment. The depth of source *z_s_* = 87 mm, the depth of acoustic sensor *z_r_* = 10 mm, the depth of water layer *H* = 182 mm. The sound speed *c*_1_ = 1450.212 m·s^−1^(obtained by the empirical formula with the water temperature in tank, the temperature is measured by the temperature recorder of Star-Oddi company [30]).

**Figure 13:**
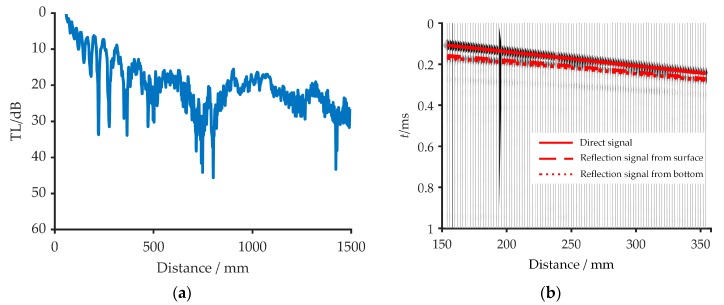
(**a**) The TL measured in the experiment; (**b**) The arrival time of the signal. The red line represents the arrival time of the direct signal, the dashed red line means the arrival time of surface reflection signal, and the dotted red line means the arrival time of the bottom reflection signal.

During the measurement experiment process, the source was fixed and transmitted the CW signal with the center frequency 135 kHz. The acoustic sensor was fixed on the movable walkway with a sampling rate at *f_s_* = 20 MHz. The starting point was 60 m away from the source position. The sampling rate of each measurement point was repeated ten times to reduce the influences of random error. After completing the one-point measurement, the walkway drives the acoustic sensor to move away from the transmitter by 2 mm, and repeated the same for 719 points. Figure 13a shows the TL measured in the experiment. Figure 13b shows the time-domain diagram of the 50th to 150th receiving signal received during the experiment. The red lines in Figure 13b represent the arrival time of the direct signal, the surface reflection signal, and the bottom reflection signal at the reception point from the 50th to the 150th. The measurement parameters are shown in Table 3. It can be inferred from Figure 13b that the arrival time of each path signal achieved by the simulation is consistent with the actual arrival time, which verifies the reliability of the measurement experiment.

### 5.3. Analysis of Inversion Results

Table 4 shows the inversion values of the seabed and search bounds of five parameters. In the processing of experimental data, the SA Settings are identical to the ones in Section 4.3.

**Figure 14:**
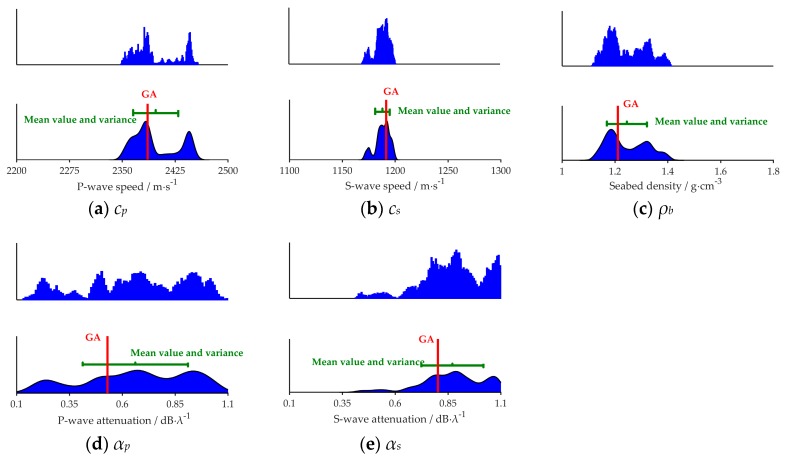
Five geo-acoustic parameters’ PPD. Red lines mean the inversion result of each parameter by GA. Green segments represent the mean value of the inversion results and their variance.

Figure 14 shows the PPDs of the five geoacoustic parameters. The vertical axis represents the probability, and GA means the inversion result of GA indicated by the red line. The mean value and variance of inversion results are indicated by the green segment in which the mean value is shown at the middle of the segment. The length of the green segment reflects the variance of each parameter. We can easily see from Figure 14 that different parameters have different sensitivities.

The PPDs of *c_p_*, *c_s_* and *ρ_b_* are relatively narrow in their prior search bounds, which proves *c_p_*, *c_s_*, *ρ_b_* are more sensitive to the cost function, and have fewer uncertainties. The PPDs of *α_p_*, *α_s_* are flat in their prior search bounds, which means that *α_p_* and *α_s_* are not sensitive to the cost function. The sharpness of the probability density curve shows the sensitivity of the cost function to every parameter: *c_p_*, *c_s_* > *ρ_b_* > *α_p_*, and *α_s_*. The inversion results obtained via the measurement experiment data are consistent with the simulation results discussed in Section 4.

**Figure 15:**
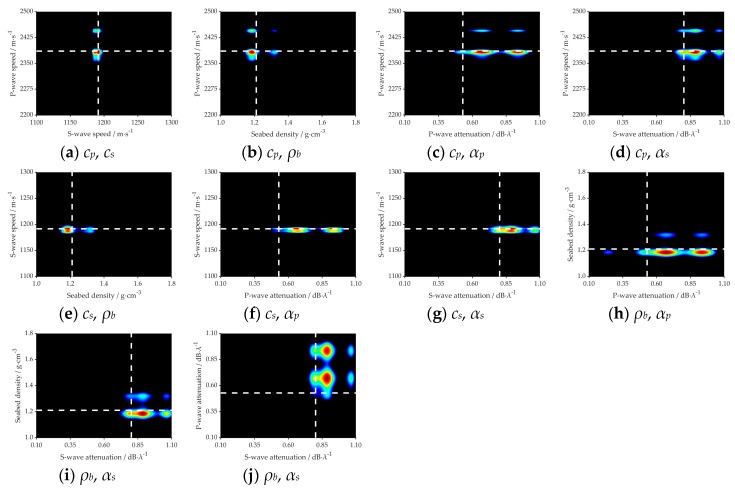
2D marginal PPDs between different parameters. White dashed lines mark the Genetic Algorithm (GA) results of each parameter.

Figure 15 represents the 2D marginal of the PPDs, where the dashed white line indicates the GA inversion result of each parameter, and the values of the variance represent the magnitude of the uncertainty. As shown in Figure 15a,b,e, the result of GA is near or at the highest probability. While in the Figure 15c,d,f–j, we can see that there is a little deviation between those two kinds of inversion methods, but for each parameter, the result of GA is within the margin of error. The density of the plastic plates was known in the measurement experiment, which was 1.2 g·cm^−3^. It is very near to the inversion value, which proves the viability of the inversion results. Thus, it is concluded that: *cp*, *cs*, and *ρb* are more sensitive and have fewer uncertainties to acoustic pressure than *αp* and *αs*.

### 5.4. The Verification

**Figure 16:**
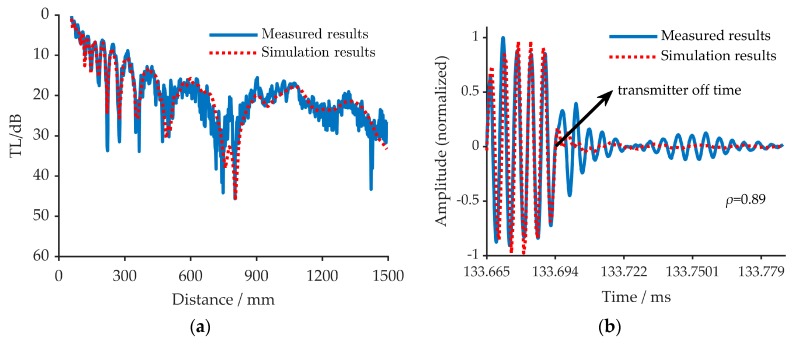
The verification approaches, (**a**) solid blue line means the TL measured in experiment and the dashed red line represents the TL simulated by inversion result; (**b**) solid blue line means the normalized signal amplitude in the time domain at the 470th reception point (one meter from the starting point), and the dashed red line represents the normalized signal amplitude in the time domain simulated by inversion result. The after off effects of the transmitter are highlighted by arrows.

To verify the effectiveness of the inversion method, two different approaches are used as shown in Figure 16a,b. Figure 16a shows the comparison of the TLs obtained through measurement and simulation. As can be seen from Figure 16a, the measurement results are consistent with the simulation results. Figure 16b represents the signal amplitude of the measurement and simulation results in the time domain. Here, the two kinds of results showed a very high correlation, which reaches 0.89 when the effect of the transmitter, after it is off, is not considered. The after off effects of the transmitter are shown in Figure 16b. Thus, the approaches used here prove that the inversion results are credible.

## 6. Conclusions

A method of geoacoustic parameter inversion is proposed based on Bayesian Theory. Considering the S-wave of the seabed has a significant impact on the propagation of low-frequency acoustic signals in the shallow sea environments. Here, the five geoacoustic parameters, namely P-wave speed (*c_p_*), S-wave speed (*c_s_*), seabed density (*ρ_b_*), p-wave attenuation (*α_p_*), and s-wave attenuation (*α_s_*) are inverted. In this paper, we used simulated data and the experimental data to verify the feasibility of the inversion method. The Posterior Probability Densities (PPDs) of the five parameters are obtained using the cost function based on the Bayesian inversion theory. The PPDs and 2D marginal PPDs provide parameter estimates and uncertainties. It is summarized that *c_p_*, *c_s_*, and *ρ_b_* in the seabed have fewer uncertainties and are more sensitive to acoustic pressure than *α_p_* and *α_s_*.

Before verification, the acoustic propagation characteristics are analyzed, which corresponded to the inversion result. Our analysis results showed that in shallow sea environments, the influence order of the degree of five parameters on acoustic propagation characteristics could be summarized as *c_p_* > *c_s_* > *ρ_b_* > *α_p_* > *α_s_*.

In the simulation, we find that the sea noise could affect the inversion results. In the noisy environment, the PPDs of every parameter will be wider than in the noiseless environment, the mean of PPD still could reflect the true values of geoacoustic parameters in simulation, and noise has less influence on the inversion results of sensitive parameters.

For further confirmation, the Transmission Loss (TL) and the normalized signal amplitude were compared, respectively, which include the measured results and the simulation results. The comparison shows that measured results are consistent with simulation results. Additionally, the density of the Poly Vinyl Chloride (PVC) plates was obtained in the measurement experiment; it was 1.2 g/m^3^, which is very close to the inversion value (1.2451 ± 0.0758g/m^3^). All these prove the credibility of the inversion method.

These results obtained from the geoacoustic inversion method will provide in-depth knowledge ‘from the basic principle to the advanced application of underwater acoustic sensors in the shallow sea acoustic propagation’ and will meet the growing interest in the need for correct geoacoustic parameter estimates and the uncertainties of the parameters.

## Figures and Tables

**Table 1:** The simulation parameters of ocean environment.

Parameters	Simulated Value
Depth *H*/m	100
Sound speed *c*_1_/m·s^−1^	1500
Sea-water density *ρ*_1_/ g·cm^−3^	1.025
P-wave speed *c_p_*/m·s^−1^	2000
S-wave speed *c_s_*/ m·s^−1^	1000
Seabed density *ρ_b_*/g·cm^−3^	1.5
P-wave attenuation *α_p_*/dB·*λ*^−1^	0.5
S-wave attenuation *α_s_*/ dB·*λ*^−1^	0.5

**Table 2:** Inversion parameter list. The true values, search bounds and inversion values are shown.

Parameters	True Values	Search Bounds	Inversion Values (Noiseless)	Inversion Values (Noisy)
*c_p_*/m·s^−1^	2000	1800, 2200	2000.9367 ± 14.5002	2001.7484 ± 25.9427
*c_s_*/m·s^−1^	1000	900, 1100	996.5609 ± 9.1982	1000.9983 ± 11.7670
*ρ_b_*/g·cm^−3^	1.5	1.35, 1.65	1.5040 ± 0.0121	1.5063 ± 0.0152
*α_p_*/dB·λ^−1^	0.5	0.45, 0.55	0.4974 ± 0.0046	0.5023 ± 0.0053
*α_s_*/dB·λ^−1^	0.5	0.45, 0.55	0.4989 ± 0.0050	0.5015 ± 0.0.0067

**Table 3:** The measurement parameters of the anechoic tank experiment.

*z_s_* /mm	*z_r_ /*mm	*H /*mm	*c*_1_*/*m·s^−1^
87	84	182	1450.212

**Table 4:** Inversion parameters: the search bounds and inversion values are shown.

Parameters	Search Bounds	Inversion Values
*c_p_*/m·s^−1^	2200–2500	2397.3563 ± 31.9997
*c_s_*/m·s^−1^	1100–1300	1187.9400 ± 6.8722
*ρ_b_*/g·cm^−3^	1.0–1.8	1.2451 ± 0.0758
*α_p_*/dB·λ^−1^	0.1–1.1	0.6616 ± 0.2489
*α_s_*/dB·λ^−1^	0.1-1.1	0.8705 ± 0.1468
